# Cytoskeletal Protein 4.1R Inhibits BCR-Mediated B-Cell Activation by Restraining AKT1 Phosphorylation

**DOI:** 10.3390/cells15141256

**Published:** 2026-07-13

**Authors:** Yuying Guo, Dandan Fan, Denghui Liu, Qi Shao, Siyao Sang, Yuting Niu, Lixiang Chen, Taotao Liang

**Affiliations:** 1 School of Life Sciences, Zhengzhou University, 100 Science Road, Zhengzhou 450001, China; guoyuying12@163.com (Y.G.); dandanfan0725@163.com (D.F.); sysang@fudan.edu.cn (S.S.); 2 Henan Institute of Medical and Pharmaceutical Sciences, Zhengzhou University, Zhengzhou 450001, China; 3 Department of Forensic Science and Technology, Zhengzhou Police University, Zhengzhou 450053, China; liudenghui@zzpu.edu.cn; 4 School of Pharmaceutical Sciences, Zhengzhou University, 100 Science Road, Zhengzhou 450001, China; zhanchen_1991@163.com; 5 State Key Laboratory of Genetics and Development of Complex Phenotypes, School of Life Sciences, Fudan University, Shanghai 200438, China; 6 School of Basic Medical Sciences, Zhongzhou Laboratory, Henan University, Kaifeng 475004, China; niuyuting@henu.edu.cn; 7 Institute of Biomedical Sciences, Henan Academy of Sciences, Zhengzhou 450046, China

**Keywords:** BCR signaling, B-cell activation, protein 4.1R, AKT1 phosphorylation

## Abstract

**Highlights:**

**What are the main findings?**

- Cytoskeletal protein 4.1R negatively regulates BCR-mediated B-cell activation.
- Phosphoproteomic analysis reveals that 4.1R deficiency prolongs AKT1 activation downstream of BCR signaling.

**What are the implications of the main findings?**

- This work identifies cytoskeletal protein 4.1R as a previously unappreciated upstream negative regulator in the BCR signaling cascade.
- The specific link between 4.1R-CD19 interaction and sustained AKT1 phosphorylation provides a new, proximal signaling explanation for the B cell hyperproliferation phenotype observed in 4.1R-deficient cells.

**Abstract:**

B-cell receptor (BCR) is indispensable for B-cell responses, and its signaling relies on the rearrangement of cytoskeletal proteins. Cytoskeletal protein 4.1R has been previously implicated in the regulation of immune function. However, the specific role of 4.1R in BCR-mediated B-cell activation remains unknown. Here, we performed single-cell RNA (scRNA) sequencing on splenic B cells isolated from wild-type (WT) and 4.1R-knockout (4.1R-KO) mice to systematically characterize the functional contribution of 4.1R to B-cell biology. Transcriptomic analyses suggested a critical role for 4.1R in modulating BCR signaling. Ex vivo stimulation of primary B cells with anti-IgM demonstrated that 4.1R-KO B cells exhibited marked overactivation, hyperproliferation, and enhanced antibody secretion. Furthermore, unbiased phosphoproteomic profiling, identified sustained AKT1 phosphorylation as a key feature in 4.1R-KO B cells. Subsequent functional validation confirmed that 4.1R regulates BCR signaling by constraining AKT1 activation. Mechanistically, 4.1R rapidly colocalized with the coreceptor CD19 at the plasma membrane upon BCR engagement, and co-immunoprecipitation confirmed their physical interaction. Loss of 4.1R disrupted this interaction and resulted in sustained and amplified AKT1 phosphorylation (but not AKT2) in stimulated B cells. Collectively, our findings identify 4.1R as a novel negative regulator of BCR signaling that interacts with CD19 to constrain AKT1 activation.

## 1. Introduction

B cells play an essential role in humoral immunity by recognizing antigens, initiating intracellular signaling cascades, and undergoing clonal expansion and differentiation [1]. Naive B cells are maintained in a quiescent state under resting conditions, marked by a small size and cell cycle arrest in G0 [2]. Upon antigen stimulation, engagement of the B-cell receptor (BCR) triggers rapid phosphorylation of proximal kinases, including SYK, LYN, et al. [3,4]. This initial signal is robustly amplified by the co-receptor CD19, which lowers the activation threshold of B cells and facilitates the recruitment of downstream signaling effectors [5,6]. CD19 signaling in turn drives AKT activation, and activated AKT negatively regulates GSK3β via phosphorylation at its Ser9 residue, ultimately inducing the metabolic and proliferative responses required for full B cell activation [7,8]. Elucidating the regulatory mechanisms underlying BCR-mediated B-cell activation is critical for advancing our understanding of humoral immunity and developing targeted therapeutic strategies for B-cell-associated disorders [9,10,11].

Cytoskeletal remodeling influences immune synapse formation, receptor clustering, and signal propagation in B cells [12]. Naive B cells primarily rely on the membrane-associated cytoskeleton to maintain their shape. In contrast, upon activation, B cells markedly increase their cytoplasmic volume through cytoskeletal rearrangement to facilitate large-scale immunoglobulin secretion [13]. For example, Ezrin, a membrane-cytoskeleton linker protein, not only regulates the formation of BCR microclusters but also modulates BCR signaling by controlling downstream ERK phosphorylation, thereby ultimately influencing B-cell responses [14,15]. 4.1R is a core member of the 4.1 family (including 4.1R [16], 4.1G [17], 4.1N [18], 4.1B [19]). It functions as a membrane-skeleton adaptor and occurs in two isoforms (135 kDa and/or 80 kDa), which result from alternative splicing of the headpiece domain [20]. 4.1R is broadly expressed in hematopoietic cells and has been implicated in cell adhesion, migration, and membrane structural stability. Recent research indicates 4.1R plays a key role in immune cell activation. For example, in T cells and mast cells, it regulates TCR and FcεRI signaling pathways by modulating LAT phosphorylation [21,22]. Our previous work has further shown that 4.1R participates in TLR4-mediated B-cell activation and regulates the fate of activated B cells [23]. However, the role of 4.1R in BCR signaling and B-cell synapse formation remains poorly understood.

Given the growing evidence linking 4.1R to cellular signaling and immune regulation, this study investigated whether 4.1R modulates BCR-mediated B-cell activation. We integrated approaches including single-cell transcriptomic profiling, genetic deletion models, functional assays (flow cytometry, CFSE-based proliferation assays, immunofluorescence staining, and co-immunoprecipitation), and phosphoproteomics. Our results demonstrated that the loss of 4.1R enhances BCR-mediated B-cell activation, proliferation, and antibody secretion. Phosphoproteomics analysis revealed a significant enrichment of AKT1-GSK3β axis signaling in 4.1R-KO B cells. Further mechanistic assays confirmed that 4.1R colocalizes with CD19 at the B-cell immunological synapse upon BCR engagement. Collectively, these findings establish 4.1R as a novel negative regulator of BCR signaling that interacts with CD19 to constrain AKT1-driven B-cell proliferation. Our results define the landscape of the B-cell phosphoproteome and identify a key signaling axis through which 4.1R regulates the exit of B cells from quiescence.

## 2. Materials and Methods

### 2.1. Mice

C57BL/6J wild-type mice (WT) were purchased from Beijing Vital River Laboratory Animal Technology Company, and 4.1R-KO mice on the C57BL/6J genetic background were provided by New York Blood Center. All mice were housed under specific pathogen-free conditions at a constant room temperature of 22–24 °C with a 12 h light/dark cycle. All mice were 6–8 weeks old and included both male and female individuals. Animals were randomly assigned to experimental groups. No animals were excluded unless they failed to meet predefined tissue quality criteria. The experimental protocols were approved by the Zhengzhou University Animal Ethics and Experimentation Committee.

### 2.2. B-Cell Preparation and Culture

Splenic B cells were purified from 6- to 8-week-old WT and 4.1R-KO mice (after isotonic erythrocyte lysis) by positive selection of cells expressing CD19 using the B-cell isolation kit (130-052-201, Miltenyi Biotec, Bergisch Gladbach, Germany), according to the manufacturer’s instructions, yielding a population of >95% B220^+^ cells (Supplementary Figure S1). The purified B cells were adjusted to a density of 5 × 10^6^ cells/mL and cultured in 5% CO_2_ at 37 °C with complete RPMI-1640 medium (12633020, Gibco, Grand Island, NY, USA) containing 10% fetal bovine serum (FBS), 1% HEPES, 1% L-glutamate, 1% penicillin/streptomycin and 0.1% 2-mercaptoethanol. To ensure that the isolation process did not result in artifactual activation, the purified B cells were rested in this medium at 37 °C for 2–4 h, to allow cellular signaling to return to a basal state, prior to any stimulation or downstream analysis. Cells or samples with low viability (<80%), insufficient cell number after isolation, or failed quality control during flow cytometry acquisition were excluded from analysis.

### 2.3. Flow Cytometry

B cells were collected at 24 h after anti-IgM (7102892100, Thermo Fisher Scientific, Waltham, MA, USA; 2.5 μg/mL) stimulation. For flow cytometry analysis, cells were stained in individual tubes for each antibody condition to avoid signal interference. Cells were washed in phosphate-buffered saline (PBS) containing 2% FBS and incubated at 4 °C for 30 min with one of the following fluorescein isothiocyanate-conjugated (FITC) antibodies: FITC anti-CD86 (105005, Biolegend, San Diego, CA, USA; 1 µg per 10^6^ cells), FITC anti-B220 (103205, Biolegend, San Diego, CA, USA; 1 µg per 10^6^ cells), and FITC anti-CD69 (104505, Biolegend, San Diego, CA, USA; 1 µg per 10^6^ cells). 7-AAD viability dye (640922, Biolegend, San Diego, CA, USA;) was added to each tube 5 min prior to sample acquisition to exclude dead cells from the analysis. After two washes in PBS containing 2% FBS, cells were analyzed by flow cytometry with a BD LSRFortessa (BD Biosciences, San Jose, CA, USA), and data were processed with the Tree Star FlowJo X 10.0.7 software (BD Biosciences, Ashland, OR, USA). During analysis, doublet cells were first excluded by gating on a plot of side scatter area (SSC-A) versus side scatter height (SSC-H). Subsequently, viable B cells were then identified by negative staining for 7-AAD (Supplementary Figure S2). Unstained B cells were used as the negative control for fluorescence background subtraction.

### 2.4. ELISA Assay

Cell culture supernatants from anti-IgM-stimulated (7102892100, Thermo Fisher Scientific, Waltham, MA, USA; 2.5 μg/mL) WT and 4.1R-KO B cells were collected at the 72 h time point, and secreted IgM levels were quantified using a commercial Mouse IgM ELISA Kit (ab133047, AbCam, Cambridge, UK). Prior to the assay, the kit was equilibrated to room temperature for 30 min, and the 1× wash buffer was freshly prepared from the provided concentrated stock solution. No additional antibody coating or antibody dilution steps were required, as the capture antibody was pre-immobilized on the 96-well microwell plates by the manufacturer. Briefly, serially diluted IgM standards, collected cell supernatants, and blank negative control wells (containing only assay diluent without any sample or standard) were added to the designated pre-coated wells, followed by the addition of the HRP-conjugated detection antibody supplied in the kit. After 1 h of incubation at 37 °C, unbound reagents were thoroughly removed through 5–6 repeated wash cycles. The TMB chromogenic substrate was then added to each well, and the plate was incubated in the dark for 10–15 min until a clear blue color gradient developed across the standard wells. The reaction was terminated by adding the provided stop solution, and the optical density of each well was measured within 15 min using a microplate reader (Molecular Devices CMax Plus, Molecular Devices, San Jose, CA, USA) at 450 nm. The final IgM concentrations in all samples were calculated by fitting the blank-subtracted OD values to a four-parameter logistic standard curve.

### 2.5. Protein Extraction and Western Blot Analysis

Total proteins were extracted from B cells collected at different time points (0, 5, 10 min) after anti-IgM (7102892100, Thermo Fisher Scientific, Waltham, MA, USA; 2.5 μg/mL) stimulation. Briefly, cell pellets were harvested by centrifugation, and total cellular proteins were subsequently extracted on ice for 30 min using RIPA lysis buffer (R0020, Solarbio, Beijing, China), and protein concentrations were quantified with a Micro BCA Protein Assay Kit (PC0020, Solarbio, Beijing, China). Equal amounts of protein (40 μg per sample) were separated by SDS-PAGE and electrophoretically transferred to polyvinylidene fluoride (PVDF) membranes (IPVH00010, Millipore, Billerica, MA, USA). Membranes were blocked with 5% non-fat milk in TBST (Tris-buffered saline with 0.1% Tween-20) for 1 h at room temperature. After blocking, membranes were incubated with the indicated primary antibodies diluted in 5% BSA in TBST overnight at 4 °C. Following three washes of 10 min each with TBST, membranes were incubated with corresponding HRP-conjugated secondary antibodies (31460, 31430; Thermo Fisher Scientific, Waltham, MA, USA; 1:10,000) (diluted in 5% non-fat milk in TBST) for 1 h at room temperature. After another three washes with TBST, target protein bands were visualized using Immobilon Western Chemiluminescent HRP Substrate (PE0010, Solarbio, Beijing, China) and imaged on a ChemiDoc imaging system (Bio-Rad, Hercules, CA, USA). Densitometric quantification of band intensities was performed using ImageJ software (version 2.16.0, National Institutes of Health, Bethesda, MD, USA).

The primary antibodies used in this study were as follows: anti-AKT1 (2938, Cell Signaling Technology, Danvers, MA, USA; 1:1000), anti-Phospho-AKT1 (9018, Cell Signaling Technology, Danvers, MA, USA; 1:1000), anti-AKT2 (2964, Cell Signaling Technology, Danvers, MA, USA; 1:1000), anti-Phospho-AKT2 (8599, Cell Signaling Technology, Danvers, MA, USA; 1:1000), anti-pan-AKT (9272, Cell Signaling Technology, Danvers, MA, USA; 1:1000), anti-Phospho-pan-AKT (9611, Cell Signaling Technology, Danvers, MA, USA; 1:1000), anti-GSK3β (12456, Cell Signaling Technology, Danvers, MA, USA; 1:1000), anti-Phospho-GSK3β (9323, Cell Signaling Technology, Danvers, MA, USA; 1:1000), anti-SYK (2712, Cell Signaling Technology, Danvers, MA, USA; 1:1000), anti-Phospho-SYK (44170, Cell Signaling Technology, Danvers, MA, USA; 1:1000), anti-LYN (2732, Cell Signaling Technology, Danvers, MA, USA; 1:1000), anti-Phospho-LYN (2731, Cell Signaling Technology, Danvers, MA, USA; 1:1000), anti-BTK (8547, Cell Signaling Technology, Danvers, MA, USA; 1:1000), anti-Phospho-BTK (87141, Cell Signaling Technology, Danvers, MA, USA; 1:1000), and anti-GAPDH (10494-1-AP, Proteintech, Rosemont, IL, USA; 1:10,000). GAPDH was detected on the same membrane after stripping, or on a parallel blot from the same gel.

### 2.6. Co-Immunoprecipitation Assay

B cells were collected at 10 min after anti-IgM (7102892100, Thermo Fisher Scientific, Waltham, MA, USA; 2.5 μg/mL) stimulation, and lysed at 4 °C for 30 min in ice-cold NP40 cell buffer (FNN0021, Thermo Fisher Scientific, Waltham, MA, USA;). The supernatant was separated by centrifugation (12,000× *g*, 10 min at 4 °C) and incubated at 4 °C overnight with the anti-4.1R (sc-166759, Santa Cruz Biotechnology, Dallas, TX, USA; 2 µg for 500 µg of total protein) or anti-CD19 (sc-373897, Santa Cruz Biotechnology, Dallas, TX, USA; 2 µg for 500 µg of total protein) and the control IgG (normal mouse IgG sc-2025, Santa Cruz Biotechnology, Dallas, TX, USA; 2 µg for 500 µg of total protein), then incubated with ProteinA/G PLUS Agarose (sc-2003, Santa Cruz Biotechnology Dallas, TX, USA;) at 4 °C for 3 h. Immunoprecipitated proteins were collected by centrifugation at 3000 rpm at 4 °C for 5 min and pellets were washed 5 times with 1 mL of ice-cold RIPA lysis buffer (R0020, Solarbio, Beijing, China). Samples were boiled for 10 min and then separated by SDS-PAGE followed by transfer onto PVDF membrane. The membrane was probed with anti-4.1R (13014-1-AP, Proteintech, Rosemont, IL, USA; 1:1000) and anti-CD19 (sc-373897, Santa Cruz Biotechnology, Dallas, TX, USA; 1:1000) respectively.

### 2.7. Immunofluorescence Microscopy

B cells were collected 10 min after anti-IgM (7102892100, Thermo Fisher Scientific, Waltham, MA, USA; 2.5 μg/mL) stimulation, adjusted to a density of 1 × 10^6^ cells/mL, cytospun onto glass slides StatSpin CytoFuge 2 (Iris Sample Processing, Westwood, MA, USA), air-dried briefly, and then fixed with 4% paraformaldehyde for 15 min at room temperature. Slides were rinsed with PBS, blocked in 1% BSA (B14, Thermo Fisher Scientific, Waltham, MA, USA) for 10 min, and co-stained with anti-4.1R (13014-1-AP, Proteintech, Rosemont, IL, USA; 1:50) and anti-CD19 (27949-1-AP, Proteintech, Rosemont, IL, USA; 1:50) primary antibodies, followed by Alexa Fluor 594 anti-mouse (A-11005, Thermo Fisher Scientific, Waltham, MA, USA; 1:100) and Alexa Fluor 488 anti-rabbit (A-11008, Thermo Fisher Scientific, Waltham, MA, USA; 1:100) secondary antibodies. Nuclei were counterstained with DAPI (C0065, Solarbio, Beijing, China), and samples were mounted in Vectashield (H-1000-10, Vector Laboratories, Newark, CA, USA). Images were acquired on an LSM 510 META confocal microscope (Carl Zeiss, Oberkochen, Germany; 100×/1.3 oil objective) using LSM 510 Meta 3.2 software. Line-profile ROIs across individual cells were defined in Zen 2.6 software (Carl Zeiss, Oberkochen, Germany) to extract paired red (4.1R) and green (CD19) fluorescence intensity values along identical spatial coordinates. Raw data were exported to GraphPad Prism 8 (GraphPad Software, San Diego, CA, USA) for analysis, and Pearson’s correlation coefficients were calculated for each cell. A minimum of 3 cells from 3 independent biological replicates were analyzed per condition, with no data excluded.

### 2.8. Carboxyfluorescein Succinimidyl Ester (CFSE) Assay

Purified immature splenic B cells (5 × 10^6^ cells) were stained with 500 µL of 5 µM CFSE solution (C34554, Thermo Fisher Scientific, Waltham, MA, USA) at 4 °C for 10 min. After washing with PBS, the cells were seeded at a density of 2 × 10^5^ cells per well in a 96-well flat-bottom plate and cultured with anti-IgM (7102892100, Thermo Fisher Scientific, Waltham, MA, USA; 2.5 µg/mL) for 72 h. For flow cytometry analysis, unstained B cells were used as a negative control, and B cells stained with CFSE for 10 min served as a positive control. Proliferation was assessed based on CFSE dilution using a flow cytometer.

### 2.9. AKT1 Inhibition Assay

A-674563 as an inhibitor of AKT1 was investigated as previously described [24,25,26]. Briefly, WT and 4.1R-KO B cells were pre-treated with A-674563 (0.4 μM) (HY-N0722, MedChemExpress, Monmouth Junction, NJ, USA) for 2 h, then stimulated with anti-IgM (7102892100, Thermo Fisher Scientific, Waltham, MA, USA; 2.5 μg/mL) for different times (0, 5, 10 min). Western blot and CFSE assay were used to measure the phosphorylation of AKT1, and B-cell proliferation, respectively.

### 2.10. Single-Cell RNA Sequencing (scRNA-Seq) Sample Preparation

WT (*n* = 3 mice) and 4.1R-KO (*n* = 3 mice) mice (6–8 weeks old) were euthanized by cervical dislocation under sterile conditions. Spleens were immediately harvested and placed in ice-cold sterile PBS. Single-cell suspensions were prepared by mechanical dissociation and enzymatic digestion using collagenase A (0.1 mg/mL) and DNase I (0.01 mg/mL) at 37 °C for 20 min. Cell suspensions were filtered through a 40 μm cell strainer, followed by red blood cell lysis (R1010, Solarbio, Beijing, China). Cells were washed with cold PBS and centrifuged at 300× *g*. Cell viability was assessed using trypan blue (IR9129, Solarbio, Beijing, China) exclusion, and viable cells were counted prior to library preparation.

### 2.11. scRNA-Seq Library Preparation and Sequencing

scRNA-Seq libraries were constructed using the DNBelab C Series Single-cell Library Preparation Kit V3.0 (940-001818-00, BGI Genomics, Shenzhen, China) following the manufacturer’s instructions. Single-cell suspensions were encapsulated into microfluidic droplets using the DNBelab C4 platform (BGI Genomics, Shenzhen, China), followed by cell lysis, reverse transcription, cDNA amplification, and barcoded library construction. Libraries were circularized using the MGIEasy Circularization Kit, and DNA nanoballs (DNBs) were generated. Sequencing was performed on the DNBSEQ-T7RS platform using the DNBSEQ-T7RS High-throughput Sequencing Kit FCL PE100 V3.0 (940-000269-00, BGI Genomics, Shenzhen, China), generating paired-end 100 bp reads.

### 2.12. Data Processing and Bioinformatic Analysis

After filtering out low-quality cells (>10% mitochondrial reads, < 500 or > 6000 detected genes), 48,074 high-quality cells (mean depth: 6119 UMIs; 14,188 WT and 33,886 4.1R-KO) were retained. Data were normalized (LogNormalize), and highly variable genes were identified (FindVariableFeatures, vst method). We performed PCA (top 30 components) and corrected for batch effects across six samples using Harmony. Clustering was performed using the Louvain algorithm and visualized by UMAP. Differential expression analysis (Wilcoxon rank-sum test) identified significant genes (adjusted *p* < 0.05, log2FC > 0.25), accounting for biological replicate structure. To perform differential expression analysis between WT and 4.1R-KO B cells while accounting for biological replicate structure and avoiding pseudoreplication, we employed a pseudobulk approach. B cells (*n* = 11,046) were isolated based on marker expression (*CD19*, *MS4A1*, *CD79A*, *CD79B*) and genes detected in ≤10 cells were removed. Analyses were conducted using Seurat [27]. Cell identities were assigned using SingleR against the MonacoImmuneData reference [28], and GO enrichment was analyzed using clusterProfiler [29,30,31].

### 2.13. Sample Preparation for Phosphoproteomics

Purified mature B cells were isolated from WT (*n* = 3 mice) and 4.1R-KO (*n* = 3 mice) mice (6–8 weeks old) using the method described above, yielding at least 1 × 10^7^ cells per mouse. B cells were stimulated with anti-IgM (7102892100, Thermo Fisher Scientific, Waltham, MA, USA; 2.5 μg/mL) for 10 min and lysed on ice in lysis buffer (8 M urea, 1% protease inhibitor cocktail, 1% phosphatase inhibitor cocktail) using a high-intensity ultrasonic processor (Scientz, Ningbo, China) with three rounds of sonication. Debris was removed by centrifugation at 12,000× *g* for 10 min at 4 °C, and the supernatant was collected. Protein concentration was determined using a BCA assay kit according to the manufacturer’s instructions. For digestion, proteins were reduced with 5 mM dithiothreitol at 56 °C for 30 min and alkylated with 11 mM iodoacetamide in the dark at room temperature for 15 min. The sample was then diluted with 100 mM TEAB to reduce the urea concentration below 2 M. Trypsin was added at a 1:50 (enzyme:protein) ratio for overnight digestion, followed by a second digestion at a 1:100 ratio for 4 h. After digestion, peptides were desalted using a Strata X C18 SPE column (Phenomenex, Torrance, CA, USA), vacuum-dried, and reconstituted in 0.5 M TEAB for subsequent TMT/iTRAQ labeling according to the manufacturer’s protocol.

### 2.14. Mass Spectrometry and Data Analysis

An equal number of peptides from each sample was pooled and fractionated by off-line basic pH reverse-phase liquid chromatography (LC). Prior to LC-MS/MS analysis, phosphopeptides were selectively enriched from each fraction using the immobilized metal affinity chromatography (IMAC) approach, as previously described [32]. Using the JUMP software, spectra were searched against the Uniprot mouse database and filtered to achieve 1% false discovery rate at the peptide level [33,34]. TMT reporter ion intensities were extracted, filtered, normalized, and corrected for batch effects across independent LC-MS/MS acquisition runs using median-centering normalization and summarized into peptide and protein quantification. For B-cell activation-related phosphoproteomic analysis, only peptide groups with no missing values across all samples were retained for subsequent Student’s *t*-test. Phosphosites exhibiting a fold change > 1.3 and a *p*-value < 0.05 were defined as significantly regulated and selected for further analysis. Principal component analysis (PCA) and Kyoto Encyclopedia of Genes and Genomes (KEGG) analysis were performed using “stats” and “clusterProfiler” R packages, respectively. Functional annotation of proteins via Clusters of Orthologous Groups/EuKaryotic Orthologous Groups (COG/KOG) classification using the EggNOG database [35,36].

### 2.15. Kinase Prediction

Using the iGPS (Group-based Prediction System) software package [37] (version 1.0, http://igps.biocuckoo.org, accessed on 20 March 2020) to predict the site-specific kinase-substrate relations for all identified phosphorylation proteins in our dataset. During the prediction, the medium threshold was chosen.

### 2.16. Statistical Analysis

All statistical analyses were conducted using GraphPad Prism 8 (GraphPad Software, San Diego, CA, USA). Data are presented as mean ± SEM. The number of independent biological replicates (*n*) is indicated in each figure legend. Data normality was tested using the Shapiro–Wilk test (3 ≤ *n* ≤ 50) or the Kolmogorov-Smirnov test (*n* > 50). Normally distributed data were analyzed by unpaired Student’s *t*-test, while non-normally distributed data were analyzed by the Mann-Whitney U test. Correlations were assessed using Pearson’s correlation test for normally distributed data. A *p*-value < 0.05 was considered statistically significant for all comparisons.

## 3. Results

### 3.1. scRNA-Seq Analysis Reveals Dysregulated BCR Signaling in 4.1R-KO Splenic B Cells

**Figure 1:**
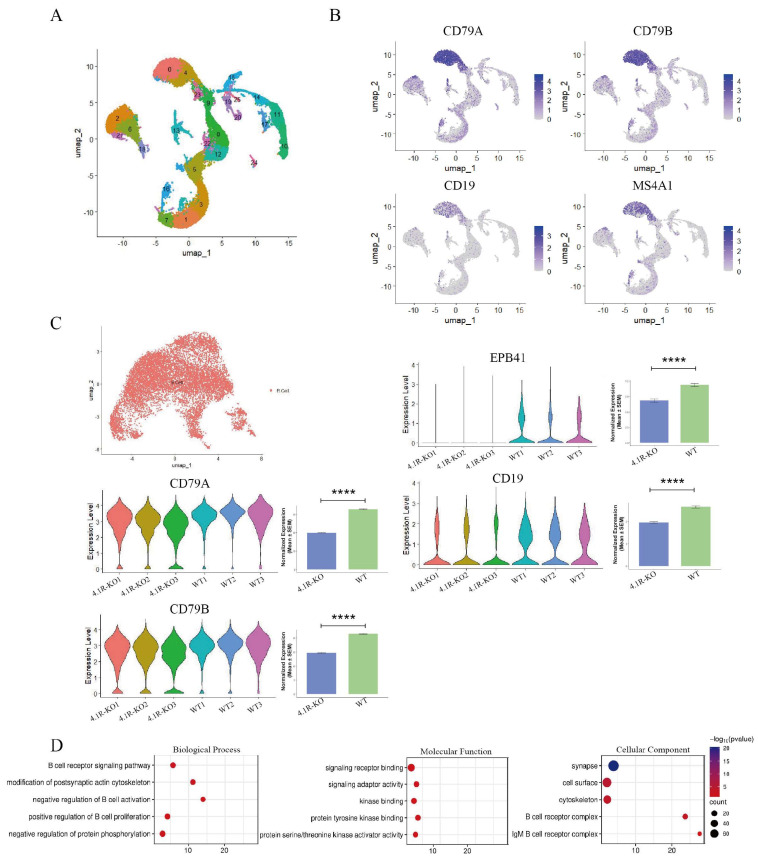
scRNA-seq analysis of spleens from WT and 4.1R-KO mice uncovers the regulatory role of 4.1R in B cells. (**A**) Uniform Manifold Approximation and Projection (UMAP) plot showing the global clustering of single-cell transcriptomes obtained from WT and 4.1R-KO mouse spleens. (**B**) The featured plot illustrates the distribution of mature B-cell markers (*CD79A*, *CD79B*, *CD19*, and *MS4A1*) across distinct clusters. (**C**) B cells were isolated from the single-cell dataset using specific markers. Violin and bar graphs depicted the statistical analysis of differential expression for *EPB41*, *CD79A*, *CD79B*, and *CD19* in B cells from WT versus 4.1R-KO mice. (**D**) GO enrichment analysis of differentially expressed genes in B cells derived from 4.1R-KO versus WT mice. A bubble plot illustrating enriched biological processes, molecular function and cellular component. All data are presented as mean ± SEM. Statistical significance was determined by unpaired Student’s *t* test. **** *p* < 0.0001.

To characterize the transcriptional consequences of 4.1R loss in mature B cells, we performed scRNA-seq on splenocytes isolated from WT and 4.1R-KO mice. Unsupervised clustering analysis divided mouse spleen cells into 26 cell subsets (Figure 1A). Based on canonical B-cell markers, including *CD79A*, *CD79B*, *CD19*, and *MS4A1*, mature B cells were identified and annotated (Figure 1B). To further determine whether 4.1R deficiency affects B-cell subset composition, we analyzed the distribution of major B-cell subsets—including follicular, marginal zone, transitional and cycling B cells—between WT and 4.1R-KO mice. No statistically significant differences in subset frequencies were observed under steady-state conditions (Supplementary Figure S3), indicating that 4.1R loss does not alter the baseline architecture of the B-cell compartment. We next extracted individual B cells from scRNA-seq data and compared gene expression patterns within the B-cell compartment between WT and 4.1R-KO mice. As expected, *EPB41* expression was selectively lost in 4.1R-KO B cells, validating the genotype. Notably, BCR-associated genes *CD79A*, *CD79B*, and *CD19* were upregulated in 4.1R-KO B cells, indicating BCR pathway transcriptional priming at the transcript level (Figure 1C). To identify signaling alterations caused by 4.1R loss in B cells, we performed functional enrichment analysis on differentially expressed genes (|Log2FC| ≥ 0.25, *p* < 0.05) (Supplementary Table S1). Pathways related to BCR signaling(*CD19*, *CD79A*, *CD79B*, *NFKB1*, *NFKBIA*, *PTPN6*) were significantly enriched in 4.1R-KO B cells (Figure 1D). Together, these findings demonstrate that 4.1R deficiency induces BCR-related transcriptional priming in B cells at steady state.

### 3.2. Loss of 4.1R Potentiates BCR-Driven B-Cell Activation, Proliferation, and Antibody Production

**Figure 2:**
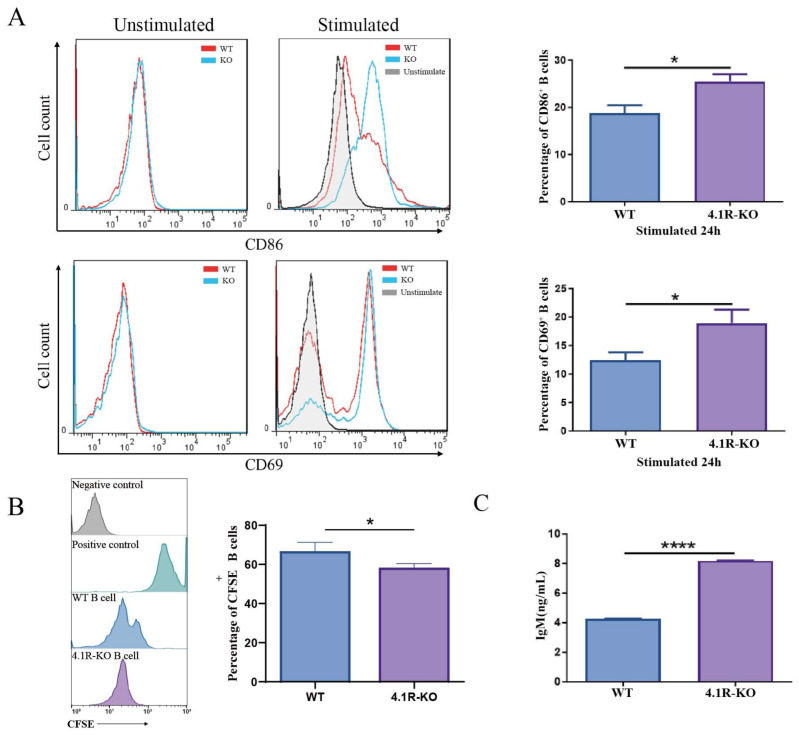
Deletion of 4.1R enhances BCR-mediated B cell activation, proliferation, and IgM secretion. (**A**) Flow cytometry analysis of CD86 (**top**) and CD69 (**bottom**) expression in WT and 4.1R-KO B cells after 24 h of stimulation with anti-IgM (2.5 μg/mL), with unstimulated cells used as a control. Red lines represent WT B cells, teal lines represent 4.1R-KO B cells, and gray shaded areas indicate unstimulated controls (*n* = 3 independent experiments). (**B**) B cell proliferation was assessed via CFSE assay. Purified WT and 4.1R-KO B cells were stimulated with anti-IgM (2.5 μg/mL) for 72 h, and cell proliferation was analyzed by flow cytometry (*n* = 3 independent experiments). (**C**) Soluble IgM levels in culture supernatants were quantified by ELISA after 72 h of stimulation (*n* = 3 independent experiments). All data are presented as mean ± SEM. Statistical significance was calculated using an unpaired two-tailed Student’s *t*-test. * *p* < 0.05; **** *p* < 0.0001.

Previous studies have shown that when encountering an antigen, B cells transition from a quiescent state to an activated state, accompanied by an increase in CD86 and CD69 expression [38,39]. To determine whether 4.1R affects BCR-mediated B-cell activation, we stimulated primary B cells with anti-IgM (2.5 μg/mL) and measured CD86 and CD69 expression by flow cytometry (Figure 2A). Results showed that the 4.1R-KO B cells exhibited significantly higher CD86 and CD69 expression levels compared with WT B cells, which suggested that 4.1R negatively regulates BCR-mediated B-cell activation. B-cell proliferation is an important event after B-cell activation. We next assessed whether 4.1R influences B-cell proliferative responses following BCR stimulation. Using a CFSE-based proliferation assay, we found that after 72 h of anti-IgM stimulation, 4.1R-KO B cells displayed a lower percentage of CFSE^+^ B cells than WT B cells, indicating enhanced proliferative capacity (Figure 2B). Given these increases in activation and proliferation-associated responses, we next examined antibody secretion. Consistent with this phenotype, 4.1R-KO B cells produced significantly higher levels of secreted IgM upon anti-IgM stimulation than WT B cells (Figure 2C). Together, these findings suggest that 4.1R acts as a negative regulator of BCR-mediated B-cell activation, proliferation, and antibody secretion, supporting a role for 4.1R in limiting early BCR signaling responses.

### 3.3. 4.1R Deficiency Remodels the BCR-Induced Phosphoproteome of Splenic B Cells

**Figure 3:**
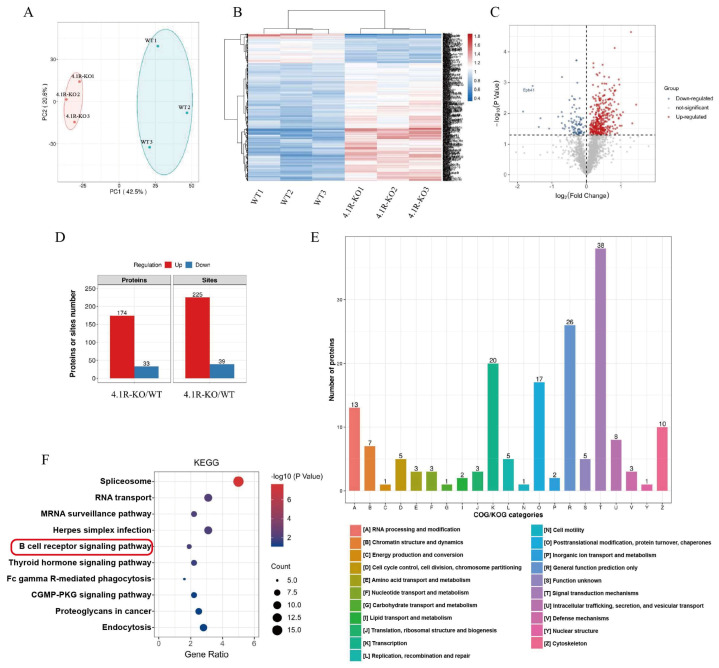
Phosphoproteomic profiling of WT (*n* = 3 mice) and 4.1R-KO (*n* = 3 mice) B cells following anti-IgM stimulation. (**A**) PCA of global phosphoproteomic profiles. (**B**) Heatmap showing hierarchical clustering of global phosphopeptide abundance in WT and 4.1R-KO B cells 10 min after stimulation with 2.5 μg/mL anti-IgM. (**C**) Volcano plot depicting significantly differentially expressed phosphopeptides between genotypes; red dots indicate upregulated phosphopeptides, and green dots indicate downregulated phosphopeptides in 4.1R-KO B cells relative to WT controls. The dashed line indicates the significance threshold of *p* < 0.05. (**D**) Bar graph summarizing the number of significantly upregulated and downregulated phosphoproteins (**left**) and phosphosites (**right**) in 4.1R-KO versus WT B cells. (**E**) COG/KOG functional classification of differentially regulated phosphopeptides. (**F**) KEGG enrichment analysis was performed for differentially phosphorylated proteins and sites.

Comparative scRNA-seq analysis of 4.1R-KO and WT spleens revealed that 4.1R primarily modulates B-cell function via BCR signaling and phosphorylation-related pathways. To further investigate the mechanism by which 4.1R regulates BCR-mediated B-cell activation and proliferation, we performed global phosphoproteomic profiling on purified splenic B cells from WT and 4.1R-KO mice stimulated with anti-IgM for 10 min. Using TMT labeling coupled with LC-MS/MS, we quantified the phosphoproteomes of stimulated B cells across three biological replicates. In total, we identified 3096 phosphorylation sites on 1646 proteins, with 2992 sites on 1593 proteins yielding quantitative data. To distinguish experimental variance from biological variation, we performed PCA on phosphopeptides (Figure 3A). The results showed clear separation between WT and 4.1R-KO samples in PCA space, while hierarchical clustering of phosphopeptides (Figure 3B) further confirmed the reproducibility of these results. Volcano plot analysis revealed numerous differentially expressed phosphorylation sites, many of which exhibited increased phosphorylation levels in 4.1R-KO cells (Figure 3C). We identified 174 up-regulated and 33 down-regulated proteins, as well as 225 up-regulated and 39 down-regulated phosphorylation sites (Figure 3D). Functional annotation of proteins corresponding to these differentially expressed phosphorylation sites, using the COG/KOG database, showed that the T group (Signal transduction mechanisms) was the most enriched category. The R (General function prediction only) and K (Translation) groups also contained relatively high numbers of proteins, whereas the O (Posttranslational modification, protein turnover, chaperones), A (RNA processing and modification), and Z (Cytoskeleton) groups were less represented (Figure 3E). Applying a 1.3-fold change cutoff, KEGG pathway analysis of the differentially phosphorylated proteins/sites revealed significant enrichment in BCR signaling pathways (Figure 3F), consistent with our functional experimental data and the previously established roles of 4.1R.

### 3.4. Prediction and Validation of AKT1 as a Key Kinase Driving Hyperproliferation in 4.1R-KO B Cells

**Figure 4:**
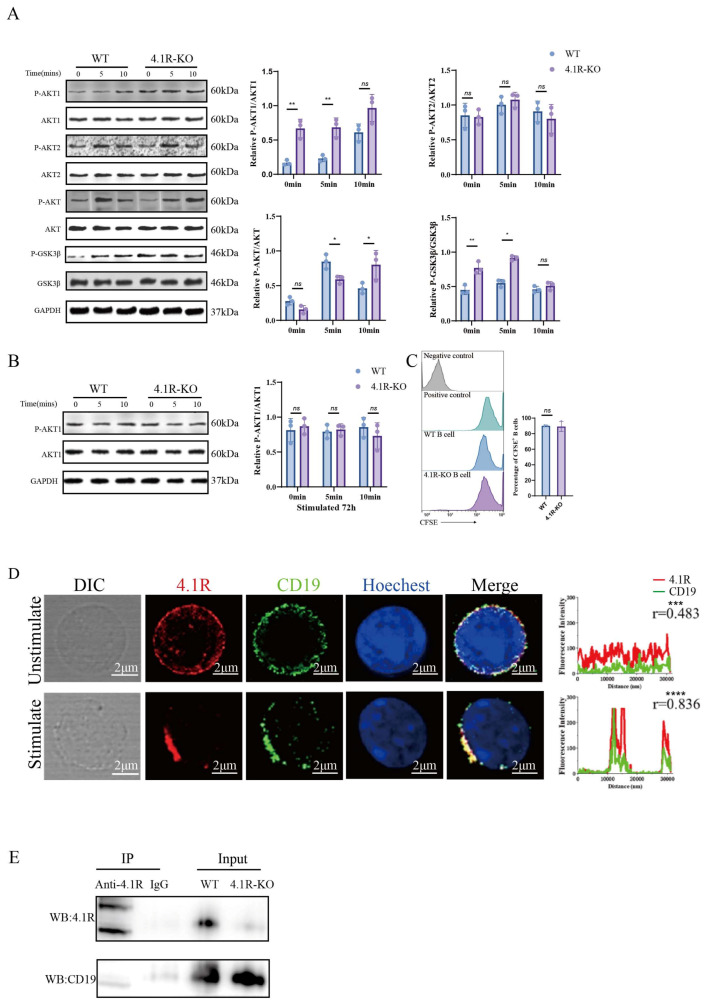
4.1R deficiency prolongs AKT1 phosphorylation in B cells. (**A**) Western blot analysis of WT and 4.1R-KO B cells stimulated with 2.5 μg/mL anti-IgM for 0, 5, and 10 min, respectively. Expression levels of phosphorylated AKT1 (P-AKT1), total AKT1, phosphorylated AKT2 (P-AKT2), total AKT2, pan-phosphorylated AKT (P-AKT), total AKT, phosphorylated GSK3β (P-GSK3β, Ser9), and total GSK3β were detected. The bar graphs to the right show densitometric quantification of relative P-AKT1/AKT1, P-AKT2/AKT2, P-AKT/AKT, and P-GSK3β/GSK3β ratios at the indicated time points in WT and 4.1R-KO B cells. Data are presented as mean ± SEM. Statistical significance was determined by Mann-Whitney U test: ns, *p* > 0.12; * *p* < 0.033; ** *p* < 0.0021. (**B**) WT and 4.1R-KO B cells were pretreated with an AKT1 inhibitor for 1 h, followed by stimulation with 2.5 μg/mL anti-IgM for 0, 5, and 10 min. P-AKT1 and AKT1 expression levels were measured by Western blot, and the bar graph shows densitometric quantification of the relative P-AKT1/AKT1 ratio. Data are presented as mean ± SEM. Statistical significance was determined by Mann-Whitney U test: ns, *p* > 0.12. (**C**) B cells were pretreated with an AKT inhibitor (0.4 µM) for 2 h. Subsequently, cells were stained with CFSE and co-stimulated with anti-IgM (2.5 µg/mL) for 72 h. Proliferation was assessed by flow cytometry, quantified as the percentage of CFSE^+^ B cells (*n* = 3 independent experiments). Data are presented as mean ± SEM. Statistical significance was calculated using an unpaired two-tailed Student’s *t*-test. ns, *p* > 0.05. (**D**) Immunofluorescence dual staining of unstimulated or 10 min anti-IgM-stimulated WT B cells was performed using anti-4.1R and anti-CD19 antibodies. Images show stimulus-dependent colocalization of 4.1R and CD19. Scale bar, 2 μm. Pearson’s correlation coefficients were calculated based on paired fluorescence intensity values of the two channels for each individual cell. At least *n* = 3 cells from three independent biological replicates were analyzed per experimental condition. The significance level of the test is set at α = 0.05. *** *p* < 0.001, **** *p* < 0.0001. (**E**) Lysates from WT and 4.1R-KO B cells were subjected to immunoprecipitation with anti-4.1R antibody or pre-immune IgG. Co-immunoprecipitation assays confirmed the interaction between 4.1R and CD19.

To identify potential kinases responsible for the observed changes in the phosphoproteome, we predicted protein kinases that may upregulate the relevant proteins (Table 1). The top 10 predicted kinases were primarily associated with the AKT, MAPK, and ERK families. While the regulatory effects of 4.1R on MAPK and ERK have been previously reported [40,41], the top-ranked kinases in our prediction also highlight AKT1, AKT2, and GSK3β as dominant candidates. Consistent with these predictions, Western blot analysis showed that 4.1R-KO B cells displayed higher and more sustained AKT1 phosphorylation than WT B cells following anti-IgM stimulation. Densitometric quantification of the accompanying bar graphs confirmed that the relative P-AKT1/AKT1 ratio was significantly increased in 4.1R-KO cells at the 5 min points, whereas P-AKT2/AKT2 showed no significant difference between the two groups. Total AKT phosphorylation was also elevated in 4.1R-KO cells, while phosphorylation of GSK3β at Ser9 was increased under the same conditions (Figure 4A). Furthermore, we applied an AKT1 phosphorylation inhibitor before stimulation, and the results showed that pharmacological inhibition of AKT1 effectively eliminated the enhanced AKT1 phosphorylation in 4.1R-KO B cells (Figure 4B). Given that 4.1R-KO B cells exhibited enhanced proliferative capacity in response to anti-IgM stimulation in Figure 2B, we next asked whether this phenotype depended on AKT1 signaling. CFSE-based proliferation analysis demonstrated that pretreatment with an AKT1 inhibitor abolished the previously observed difference in proliferative capacity between WT and 4.1R-KO B cells following anti-IgM stimulation (Figure 4C). Together, these findings indicate that the enhanced proliferative response observed in 4.1R-KO B cells is associated with sustained AKT1 phosphorylation.

### 3.5. Co-Localization of 4.1R and CD19 in B Cells

Given the critical role of CD19 in AKT signaling and our above scRNA-seq analysis showing increased CD19 expression in 4.1R-KO splenic B cells, we hypothesized that 4.1R might be a key molecule regulating CD19 membrane localization. Immunofluorescence results revealed that in unstimulated WT B cells, 4.1R and CD19 exhibited weak association, whereas upon anti-IgM stimulation for 10 min, their co-localization was significantly enhanced at the plasma membrane. Quantitative intensity analysis further confirmed a significant increase in CD19-4.1R co-localization post-stimulation, supporting a structural role for 4.1R in stabilizing CD19 microdomains (Figure 4D). Co-immunoprecipitation assays validated the interaction between 4.1R and CD19 in WT B cells, which was absent in 4.1R-KO cells (Figure 4E). These findings suggest that 4.1R may facilitate the membrane localization or clustering of CD19 upon BCR engagement.

## 4. Discussion

Our previous studies have demonstrated that protein 4.1R exerts a negative regulatory effect on LPS-induced B-cell activation. LPS triggers B-cell activation by binding to TLR4 and BCR on B cells via its polysaccharide and lipid A moieties, respectively. Our prior work focused on the regulatory role of 4.1R in the NF-κB signaling pathway downstream of TLR4. It is noteworthy that our investigation has also established that 4.1R deficiency impairs plasma cell differentiation and antibody class switching, highlighting its broad role in B cell fate determination [23]. However, the specific role and underlying mechanism of 4.1R in BCR-mediated B-cell activation remain elusive. Given the critical function of BCR in B-cell activation and effector functions, as well as the insights from our previous research, in the present study, we employed an integrated strategy combining multi-omics analyses and functional assays to investigate the role of 4.1R in B-cell biology. We further delineate a potential novel regulatory mechanism wherein the physical interaction between 4.1R and CD19 is associated with the modulation of downstream AKT1 phosphorylation and BCR-mediated B-cell activation and proliferation. This newly identified early signaling axis—wherein 4.1R constrains CD19-mediated AKT1 activation—provides a plausible upstream mechanistic explanation for the aberrant B cell proliferation observed here and may contribute to the terminal differentiation defects reported in our earlier work.

scRNA-seq analysis of resting B cells indicated that 4.1R deficiency induces a transcriptional program, which is consistent with previous reports [42]. In B cells, the 4.1R deficiency-induced transcription is characterized by the upregulation of core BCR components (including CD79A and CD79B) as well as the enrichment of the BCR signaling pathway. Notably, the expression of CD19, a co-receptor of BCR, is also increased. These transcriptional changes strongly suggest that 4.1R most likely regulates BCR-mediated processes. Subsequent functional experiments further validated this hypothesis, demonstrating that 4.1R-KO B cells exhibit enhanced activation and proliferation in response to anti-IgM stimulation. Notably, initial phosphoprotein analysis of proximal BCR signaling molecules (such as BTK, LYN, and SYK) did not reveal significant differences between WT and 4.1R-KO B cells (Supplementary Figure S4), prompting us to employ an unbiased phosphoproteomic screen. This approach ultimately identified sustained AKT1 phosphorylation as a key distinguishing feature, suggesting that 4.1R may exert its regulatory effect at a signaling node downstream of the immediate proximal cascade. This observation aligns with previous reports highlighting the negative regulatory role of 4.1R in immune cell membrane receptor signal transduction [16,21,23].

The AKT signaling pathway acts as a core regulator of cell proliferation. While prior work has demonstrated that 4.1R modulates AKT signaling [41], whether 4.1R targets a specific isoform of the AKT family remains unexplored. In this study, we confirm this regulatory relationship via global phosphoproteomic profiling, which uncovered widespread remodeling of the B cell phosphoproteome in the absence of 4.1R. More importantly, our kinase prediction and functional validation identified AKT1—the AKT family isoform—as the key mediator driving the hyperproliferative phenotype: 4.1R knockout (4.1R-KO) B cells retain sustained AKT1 phosphorylation following BCR stimulation, while pharmacological blockade of AKT1 completely abolishes this excessive proliferative response. Collectively, our results not only add independent confirmation that 4.1R acts as an upstream regulator of the AKT pathway, but also refine this model by demonstrating that 4.1R specifically regulates AKT1, not other AKT family members. It is important to note that the conclusion regarding AKT1’s central role relies primarily on pharmacological inhibition using A-674563, a selective AKT1 inhibitor. While A-674563 is widely used, potential off-target effects inherent to pharmacological tools cannot be entirely excluded. Future studies employing genetic approaches, such as AKT1 knockdown or conditional knockout in B cells, will be crucial to definitively establish the causal relationship between 4.1R deficiency, sustained AKT1 activation, and the resultant hyperproliferative phenotype. Additionally, the precise molecular mechanism by which 4.1R constrains AKT1 activation—whether through direct interaction with CD19 or via other intermediary adaptors—warrants further investigation.

CD19 is the principal BCR co-receptor that amplifies antigen-induced signaling by promoting AKT phosphorylation [43], and thus, its altered membrane distribution can markedly reshape B-cell activation thresholds [44,45]. In 4.1R-KO B cells, we observed sustained phosphorylation of AKT1. As the dominant AKT isoform driving B-cell growth and proliferation, activated AKT1 phosphorylates and thereby inhibits its key downstream substrate, GSK3β, at the Ser9 site. This AKT1-mediated inhibitory phosphorylation of GSK3β ultimately promotes increased B cell proliferation. We further demonstrate that 4.1R is rapidly recruited to the plasma membrane upon BCR engagement, where it colocalizes and physically interacts with CD19. Loss of 4.1R disrupts this association, which may lead to altered CD19 membrane dynamics or clustering, thereby contributing to potentiated AKT1 activation. The selective hyperphosphorylation of AKT1, but not AKT2, underscores the specificity of 4.1R for the AKT1-dependent metabolic and proliferative branch of BCR signaling [46]. Our results may to some extent explain the possible mechanism by which 4.1R regulates the AKT1 signaling pathway, but they do not establish a direct causal relationship between the 4.1R-CD19 interaction and AKT1 activation. It is highly likely that other molecules or signaling pathways are involved in this process, which warrants further exploration through recent literature and the application of the latest technological approaches.

Established cytoskeleton-associated proteins, such as Ezrin and Moesin, link the plasma membrane to the cortical actin cytoskeleton via their ERM domains, and play critical roles in BCR clustering, immunological synapse formation, and sustained signaling. For instance, phosphorylation of Ezrin promotes its colocalization with the BCR and enhances downstream ERK pathway activation; by contrast, Moesin modulates BCR lateral aggregation by regulating membrane domain fluidity [47,48]. The 4.1R-dependent phosphorylation changes identified in this study, particularly the altered AKT1 activity, suggest that 4.1R may act as a regulatory molecule that indirectly controls the recruitment and activation kinetics of these kinases by influencing the tension or organization of cytoskeleton-plasma membrane junctions. Unlike Ezrin/Moesin, which primarily drive cortical actin remodeling, 4.1R appears to preferentially stabilize the spatial conformation of the BCR signaling complex through interactions between its FERM domain and transmembrane proteins (e.g., CD19) as well as submembrane cytoskeletal proteins (e.g., spectrin), thereby modulating the phosphorylation status of downstream kinases. Furthermore, LAT (Linker for Activation of T cells) and its associated proteins have been well characterized as key signaling scaffolds in T cells, and previous studies have reported that 4.1R suppresses T cell activation by inhibiting LAT phosphorylation [21]. The observed alterations in AKT1/GSK3β phosphorylation under 4.1R-deficient conditions in this study indicate that 4.1R may exert a LAT-like scaffolding function, governing the specificity and strength of signaling pathways by orchestrating the spatial proximity between specific kinases and their substrates. Future investigations can further explore whether 4.1R interacts with known B cell scaffolding proteins (e.g., BLNK), or modulates the colocalization of signaling proteins by regulating the stability of lipid raft membrane microdomains.

A limitation of this study is that the functional validation was performed ex vivo primarily. While our integrated analysis of scRNA-Seq data did not reveal major alterations in steady-state B-cell subsets, our findings are based on studies in healthy mouse models and have not been validated in autoimmune disease contexts such as rheumatoid arthritis. Future studies employing in vivo models—including immunization with T-dependent antigens, detailed analysis of germinal center reactions, and investigation in autoimmune-prone or disease-specific mouse models—will be crucial to fully understand the physiological and translational implications of 4.1R-mediated regulation of the CD19-AKT1 axis in humoral immune responses under both normal and pathological conditions.

In summary, our study identified 4.1R as a key negative regulatory factor for B-cell activation, integrating transcription, phosphoproteomics, and functional analysis to propose a new regulatory axis. These findings not only deepen our understanding of B-cell homeostasis, but also provide potential therapeutic targets for treating B-cell-mediated immune dysfunction.

## Figures and Tables

**Table 1:** Top 10 predicted kinases identified from phosphoproteomic datasets using the iGPS webtool.

Entry Name	Protein Names	Gene Names	SsKSR	KSR	TF Family
AKT1_MOUSE	RAC-alpha serne/threomne-protemn kimase (EC 2.7.11.1) (AKT1 knase) (Protemn kinase B) (PKB)(Protein kinase B alpha) (PKB alpha) (Proto-oncogene c-Akt) (RAC-PK-alpha) (Thymoma viralproto-oncogene)	*Akt1 Akt Rac*	45	36	N/A
GSK3B_MOUSE	Glycogen synthase kinase-3 beta (GSK-3 beta) (EC 2.7.11.26) (Serine/threonine-protein kinaseGSK3B) (EC2.7.11.1)	*Gsk3b*	28	25	N/A
KS6B1_MOUSE	Ribosomal protein S6 kinase beta-1 (S6K-beta-1) (S6K1) (EC 2.7.11 1) (70 kDa ribosomal protein S6 kinase 1) (P70SGK1) (p70-S6K1) (Ribosomal protein S6 kinase I) (S6K) (p70 ribosomal S6 kinase alpha) (p70 S6 kinase alpha) (p70 S6K-alpha) (p70 S6KA)	*Rps6kbl*	27	20	N/A
MK14_MOUSE	Mitogen-activated protein kinase 14 (MAP kinase 14) (MAPK 14) (EC 2.7.11.24) (CRK1) (Mitogen-activated protein kinase p38 alpha) (MAP kinase p38 alpha)	*Mapk14 Crkl Csbpl Csbp2*	26	24	N/A
MK03_MOUSE	Mitogen-activated protein kinase 3 (MAP kinase 3) (MAPK 3) (EC 2.7.11.24) (ERT2) (Extracellular signal-regulated kinase 1) (ERK-1) (Insulin-stimulated MAP2 kinase) (MAP kinase isoform p44) (p44-MAPK) (MNK1) (Microtubule-associated protein 2 kinase) (p44-ERK1)	*Mapk3 Erkl Prkm3*	24	23	N/A
AKT2_MOUSE	RAC-beta serine/threonine-protein kinase (EC 2.7.11.1) (Protein kinase Akt-2) (Protein kinase B beta) (PKB beta) (RAC-PK-beta)	*Akt2*	23	19	N/A
KPCB_MOUSE	Protein kinase C beta type (PKC-B) (PKC-beta) (EC 2.7.11.13)	*Prkcb Pkcb Prkcbl*	22	18	N/A
Q4VA93_MOUSE	Protein kinase C (EC 2.7.11.13)	*Prkca mCG_140727*	21	18	N/A
ROCK1_MOUSE	Rho-associated protein kinase 1 (EC2.7.11.1) (Rho-associated, coiled-coil-containing protein kinase 1) (Rho-associated, coiled-coil-containing protein kinase I) (ROCK-I) (p160 ROCK-1) (p160ROCK)	*Rockl*	21	14	N/A
STK11_MOUSE	Serine/threonine-protein kinase STK11 (EC 2.7.11.1) (Liver kinase B1 homolog) (LKB1) (mLKB1)	*Stk11 Lkb1*	20	20	N/A

## Data Availability

The mass spectrometry proteomics data generated in this study have been deposited to the ProteomeXchange Consortium via the iProX partner repository with the dataset identifier PXD071748 (iProX ID: IPX0014593000). The scRNA-seq dataset analyzed in this study is available in the China National GeneBank DataBase (CNGBdb) under accession number CNP0008586. Additional data supporting the findings of this study are available from the corresponding author upon reasonable request.

## Supplementary Materials

The following supporting information can be downloaded at: https://www.mdpi.com/article/10.3390/cells15141256/s1, Figure S1: Flow cytometry analysis of splenic cells from wild-type (WT) mouse before and after CD19^+^ B cell isolation; Figure S2: Gating strategy for flow cytometric analysis of mouse splenic B cells; Figure S3: 4.1R does not regulate the differentiation and development of mouse B cells; Figure S4: 4.1R deficiency does not impair early BCR signaling kinase phosphorylation; Table S1: Differential gene expression analysis between WT and 4.1R-KO B cells.

## Abbreviations

The following abbreviations are used in this manuscript:

BCR	B-cell receptor
scRNA	single-cell RNA
4.1R-KO	4.1R-knockout
WT	Wild-type
FBS	Fetal bovine serum
PBS	Phosphate-buffered saline
FITC	Fluorescein isothiocyanate-conjugated
PVDF	Polyvinylidene difluoride
CFSE	Carboxyfluorescein succinimidyl ester
GO	Gene Ontology
KEGG	Kyoto Encyclopedia of Genes and Genomes
LC	Liquid chromatography
PCA	Principal component analysis
COG/KOG	Clusters of Orthologous Groups/EuKaryotic Orthologous Groups
iGPS	Group-based Prediction System

## References

[1] Tanaka S., Baba Y.B. (2020). Cell Receptor Signaling. Adv. Exp. Med. Biol..

[2] Inoue T., Shinnakasu R., Kawai C., Ise W., Kawakami E., Sax N., Oki T., Kitamura T., Yamashita K., Fukuyama H. (2021). Exit from germinal center to become quiescent memory B cells depends on metabolic reprograming and provision of a survival signal. J. Exp. Med..

[3] Song W., Kim T.J., Liu W. (2025). Editorial: Community series in BCR signaling and B cell activation, volume 2. Front. Immunol..

[4] Fiske B.E., Wemlinger S.M., Crute B.W., Getahun A. (2026). Lyn governs the establishment and maintenance of B cell anergy by suppressing PI3K signaling. Nat. Commun..

[5] Li X., Ding Y., Zi M., Sun L., Zhang W., Chen S., Xu Y. (2017). CD19, from bench to bedside. Immunol. Lett..

[6] Xu Y., Fairfax K., Light A., Huntington N.D., Tarlinton D.M. (2014). CD19 differentially regulates BCR signalling through the recruitment of PI3K. Autoimmunity.

[7] Atisha-Fregoso Y., Toz B., Diamond B. (2021). Meant to B: B cells as a therapeutic target in systemic lupus erythematosus. J. Clin. Investig..

[8] Wen Y., Jing Y., Yang L., Kang D., Jiang P., Li N., Cheng J., Li J., Li X., Peng Z. (2019). The regulators of BCR signaling during B cell activation. Blood Sci..

[9] Zhu Y., Gao L., Han Y., Liu F., Xie X., Dai X., Wang Y., Guo Y., Luo C., Chen Y. (2026). B cell receptor signaling in autoimmune rheumatic diseases: Regulatory mechanisms and therapeutic targeting. Front. Immunol..

[10] Kheirallah S., Caron P., Gross E., Quillet-Mary A., Bertrand-Michel J., Fournié J.-J., Laurent G., Bezombes C. (2010). Rituximab inhibits B-cell receptor signaling. Blood.

[11] Gómez Hernández G., Morell M., Alarcón-Riquelme M.E. (2021). The Role of BANK1 in B Cell Signaling and Disease. Cells.

[12] Pethe A., Hartmann T.N. (2025). The cytoskeletal control of B cell receptor and integrin signaling in normal B cells and chronic lymphocytic leukemia. FEBS Lett..

[13] Bhanja A., Rey-Suarez I., Song W., Upadhyaya A. (2022). Bidirectional feedback between BCR signaling and actin cytoskeletal dynamics. FEBS J..

[14] Treanor B., Depoil D., Bruckbauer A., Batista F.D. (2011). Dynamic cortical actin remodeling by ERM proteins controls BCR microcluster organization and integrity. J. Exp. Med..

[15] Batista F., Treanor B., Harwood N. (2010). Visualizing a role for the actin cytoskeleton in the regulation of B-cell activation. Immunol. Rev..

[16] Liu J., Ding C., Liu X., Kang Q. (2024). Cytoskeletal Protein 4.1R in Health and Diseases. Biomolecules.

[17] Sanuki R., Watanabe S., Sugita Y., Irie S., Kozuka T., Shimada M., Ueno S., Usukura J., Furukawa T. (2015). Protein-4.1G-Mediated Membrane Trafficking Is Essential for Correct Rod Synaptic Location in the Retina and for Normal Visual Function. Cell Rep..

[18] Pushkin A.N., Kay Y., Herring B.E. (2023). Protein 4.1 N Plays a Cell Type-Specific Role in Hippocampal Glutamatergic Synapse Regulation. J. Neurosci..

[19] Wong S.Y., Haack H., Kissil J.L., Barry M., Bronson R.T., Shen S.S., Whittaker C.A., Crowley D., Hynes R.O. (2007). Protein 4.1 B suppresses prostate cancer progression and metastasis. Proc. Natl. Acad. Sci. USA.

[20] Hoover K.B., Bryant P.J. (2000). The genetics of the protein 4.1 family: Organizers of the membrane and cytoskeleton. Curr. Opin. Cell Biol..

[21] Kang Q., Yu Y., Pei X., Hughes R., Heck S., Zhang X., Guo X., Halverson G., Mohandas N., An X. (2009). Cytoskeletal protein 4.1R negatively regulates T-cell activation by inhibiting the phosphorylation of LAT. Blood.

[22] Draberova L., Draberova H., Potuckova L., Halova I., Bambouskova M., Mohandas N., Draber P. (2019). Cytoskeletal Protein 4.1R Is a Positive Regulator of the FcεRI Signaling and Chemotaxis in Mast Cells. Front. Immunol..

[23] Liang T., Guo Y., Li M., Ding C., Sang S., Zhou T., Shao Q., Liu X., Lu J., Ji Z. (2020). Cytoskeleton protein 4.1R regulates B-cell fate by modulating the canonical NF-κB pathway. Immunology.

[24] Chorner P.M., Moorehead R.A. (2018). A-674563, a putative AKT1 inhibitor that also suppresses CDK2 activity, inhibits human NSCLC cell growth more effectively than the pan-AKT inhibitor, MK-2206. PLoS ONE.

[25] Xu L., Zhang Y., Gao M., Wang G., Fu Y. (2016). Concurrent targeting Akt and sphingosine kinase 1 by A-674563 in acute myeloid leukemia cells. Biochem. Biophys. Res. Commun..

[26] Hirai T., Wang W., Murono N., Iwasa K., Inoue M. (2024). Potential role of Akt in the regulation of fibroblast growth factor 21 by berberine. J. Nat. Med..

[27] Gribov A., Sill M., Lück S., Rücker F., Döhner K., Bullinger L., Benner A., Unwin A. (2010). SEURAT: Visual analytics for the integrated analysis of microarray data. BMC Med. Genom..

[28] Aran D., Looney A.P., Liu L., Wu E., Fong V., Hsu A., Chak S., Naikawadi R.P., Wolters P.J., Abate A.R. (2019). Reference-based analysis of lung single-cell sequencing reveals a transitional profibrotic macrophage. Nat. Immunol..

[29] Yu G., Wang L.-G., Han Y., He Q.-Y. (2012). clusterProfiler: An R package for comparing biological themes among gene clusters. OMICS.

[30] Kanehisa M., Furumichi M., Sato Y., Kawashima M., Ishiguro-Watanabe M. (2023). KEGG for taxonomy-based analysis of pathways and genomes. Nucleic Acids Res..

[31] Aleksander S.A., Balhoff J., Carbon S., Cherry J.M., Drabkin H.J., Ebert D., Feuermann M., Gaudet P., Harris N.L., Hill D.P. (2023). The Gene Ontology knowledgebase in 2023. Genetics.

[32] Tan H., Wu Z., Wang H., Bai B., Li Y., Wang X., Zhai B., Beach T.G., Peng J. (2015). Refined phosphopeptide enrichment by phosphate additive and the analysis of human brain phosphoproteome. Proteomics.

[33] Li Y., Wang X., Cho J.H., Shaw T.I., Wu Z., Bai B., Wang H., Zhou S., Beach T.G., Wu G. (2016). JUMPg: An Integrative Proteogenomics Pipeline Identifying Unannotated Proteins in Human Brain and Cancer Cells. J. Proteome Res..

[34] Wang X., Li Y., Wu Z., Wang H., Tan H., Peng J. (2014). JUMP: A Tag-based Database Search Tool for Peptide Identification with High Sensitivity and Accuracy. Mol. Cell. Proteom..

[35] Galperin M.Y., Wolf Y.I., Makarova K.S., Vera Alvarez R., Landsman D., Koonin E.V. (2021). COG database update: Focus on microbial diversity, model organisms, and widespread pathogens. Nucleic Acids Res..

[36] Hernández-Plaza A., Szklarczyk D., Botas J., Cantalapiedra Carlos P., Giner-Lamia J., Mende D.R., Kirsch R., Rattei T., Letunic I., Jensen Lars J. (2023). eggNOG 6.0: Enabling comparative genomics across 12,535 organisms. Nucleic Acids Res..

[37] Chen M., Zhang W., Gou Y., Xu D., Wei Y., Liu D., Han C., Huang X., Li C., Ning W. (2023). GPS 6.0: An updated server for prediction of kinase-specific phosphorylation sites in proteins. Nucleic Acids Res..

[38] Axelsson S., Magnuson A., Lange A., Alshamari A., Hörnquist E.H., Hultgren O. (2020). A combination of the activation marker CD86 and the immune checkpoint marker B and T lymphocyte attenuator (BTLA) indicates a putative permissive activation state of B cell subtypes in healthy blood donors independent of age and sex. BMC Immunol..

[39] Erlanson M., Grönlund E., Löfvenberg E., Roos G., Lindh J. (1998). Expression of activation markers CD23 and CD69 in B-cell non-Hodgkin’s lymphoma. Eur. J. Haematol..

[40] Breig O., Théoleyre-Schaal O., Baklouti F. (2013). Combined inhibition of PI3K and activation of MAPK p38 signaling pathways trigger erythroid alternative splicing switch of 4.1R pre-mRNA in DMSO-induced erythroleukemia cells. Cell. Signal..

[41] Chen L., Wang T., Ji X., Ding C., Liang T., Liu X., Lu J., Guo X., Kang Q., Ji Z. (2019). Cytoskeleton protein 4.1R suppresses murine keratinocyte cell hyperproliferation via activating the Akt/ERK pathway in an EGFR-dependent manner. Exp. Cell Res..

[42] Zhao L., Li B., Li H., Yang K., Zhang Y., Zhang Y., Yang Y., Guo S., Fan D., Ji Z. (2026). Protein 4.1R regulates CCDC26 and impacts myeloid leukemia progression. Cell. Signal..

[43] McCaleb M.R., Miranda A.M., Ratliff K.C., Torres R.M., Pelanda R. (2023). CD19 Is Internalized Together with IgM in Proportion to B Cell Receptor Stimulation and Is Modulated by Phosphatidylinositol 3-Kinase in Bone Marrow Immature B Cells. Immunohorizons.

[44] Otero D.C., Omori S.A., Rickert R.C. (2001). CD19-dependent Activation of Akt Kinase in B-lymphocytes. J. Biol. Chem..

[45] Mohammad D.K., Nore B.F., Gustafsson M.O., Mohamed A.J., Smith C.I.E. (2016). Protein kinase B (AKT) regulates SYK activity and shuttling through 14-3-3 and importin 7. Int. J. Biochem. Cell Biol..

[46] Calamito M., Juntilla M.M., Thomas M., Northrup D.L., Rathmell J., Birnbaum M.J., Koretzky G., Allman D. (2010). Akt1 and Akt2 promote peripheral B-cell maturation and survival. Blood.

[47] Schmidt C., Christian L., Smith T.A., Tidwell J., Kim D., Tucker H.O. (2021). Lipid Rafts Interaction of the ARID3A Transcription Factor with EZRIN and G-Actin Regulates B-Cell Receptor Signaling. Diseases.

[48] Liu Q., Zhang A., Bai Y., Yang X., Liu X., Yang L., Ying Y., Luo X., Fang F., Liu C. (2025). The Immunodeficiency Profile of Lymphocytes in the Patient with Moesin Gene Mutation During Different Infection. J. Clin. Immunol..

