# Supplementary figures and images for: Cytoskeletal Protein 4.1R Inhibits BCR-Mediated B-Cell Activation by Restraining AKT1 Phosphorylation
Source: Cells. 2026 Jul 13;15(14):1256. doi: 10.3390/cells15141256 (PMC13406958; doi:10.3390/cells15141256)

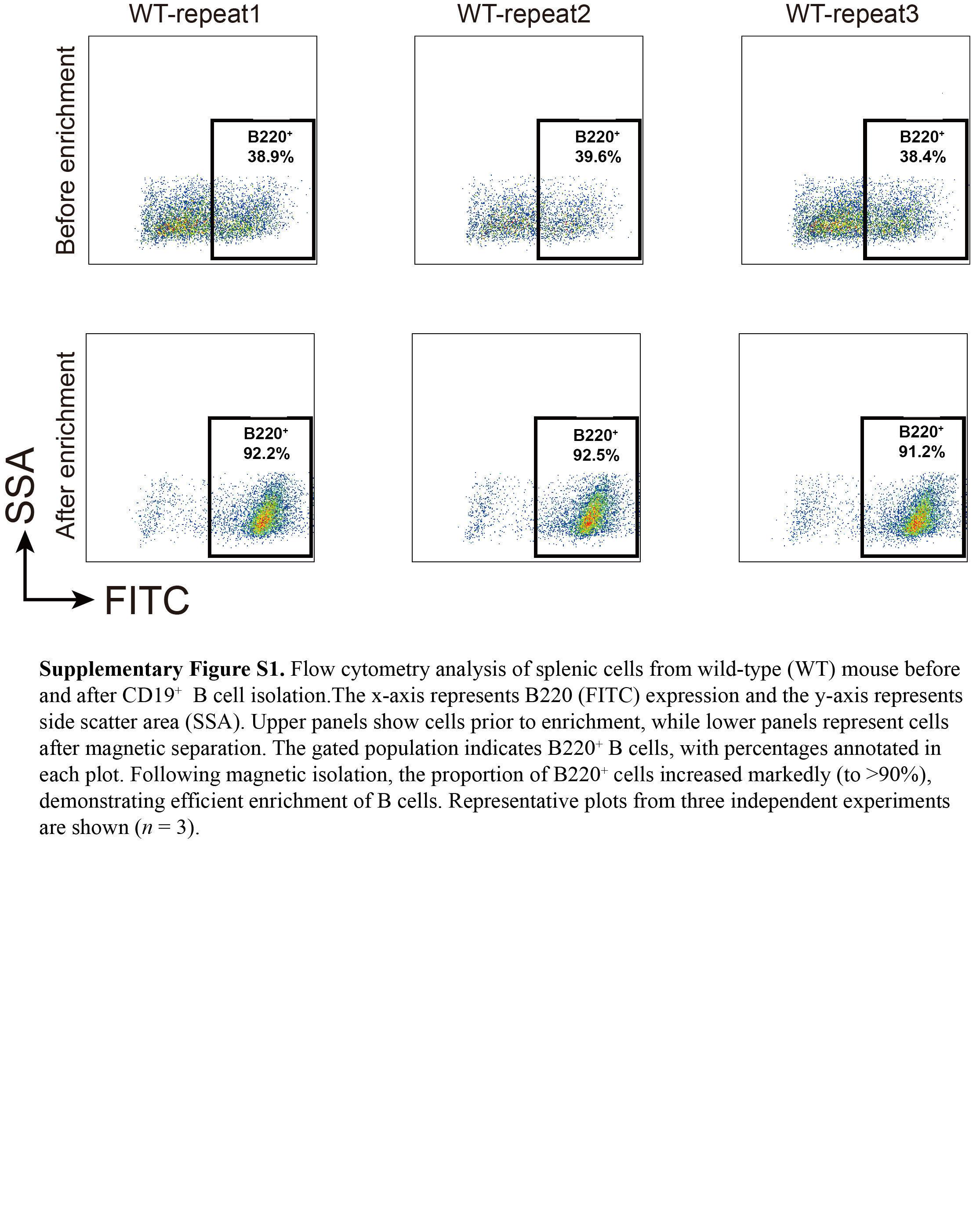

Supplement: Supplementary file 1 [file cells-15-01256-s001.zip › Figure S1.png]

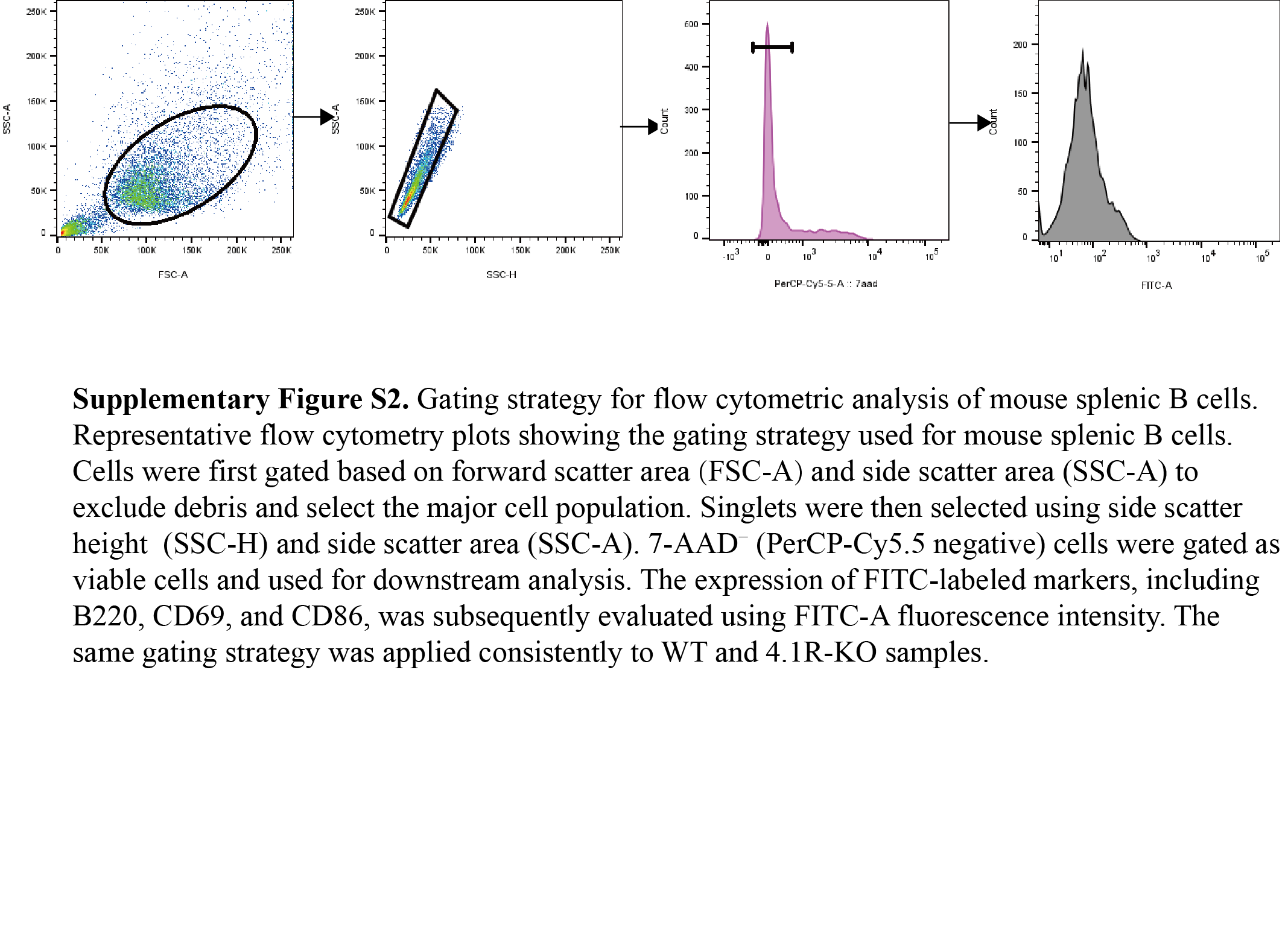

Supplement: Supplementary file 1 [file cells-15-01256-s001.zip › Figure S2.png]

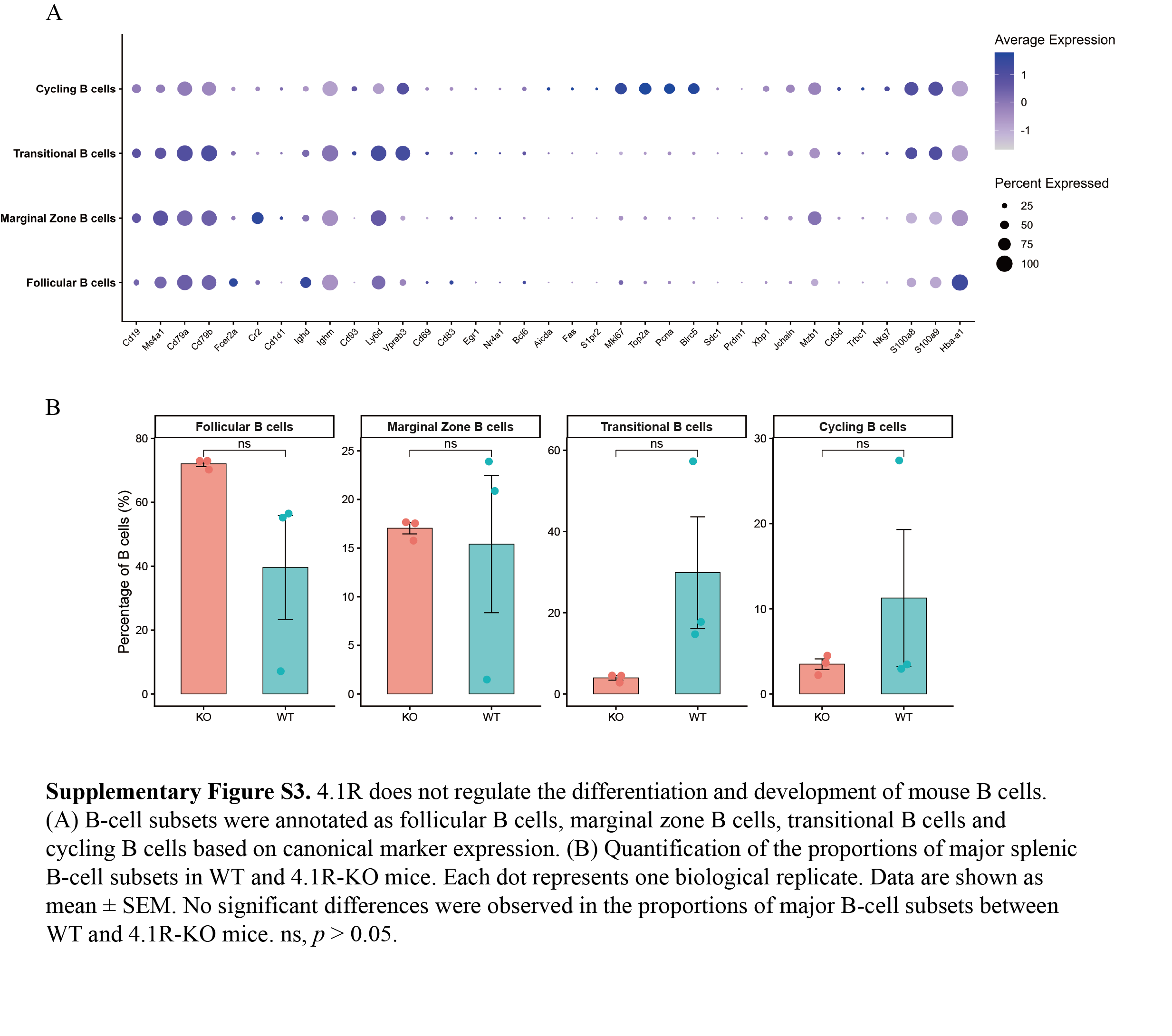

Supplement: Supplementary file 1 [file cells-15-01256-s001.zip › Figure S3.png]

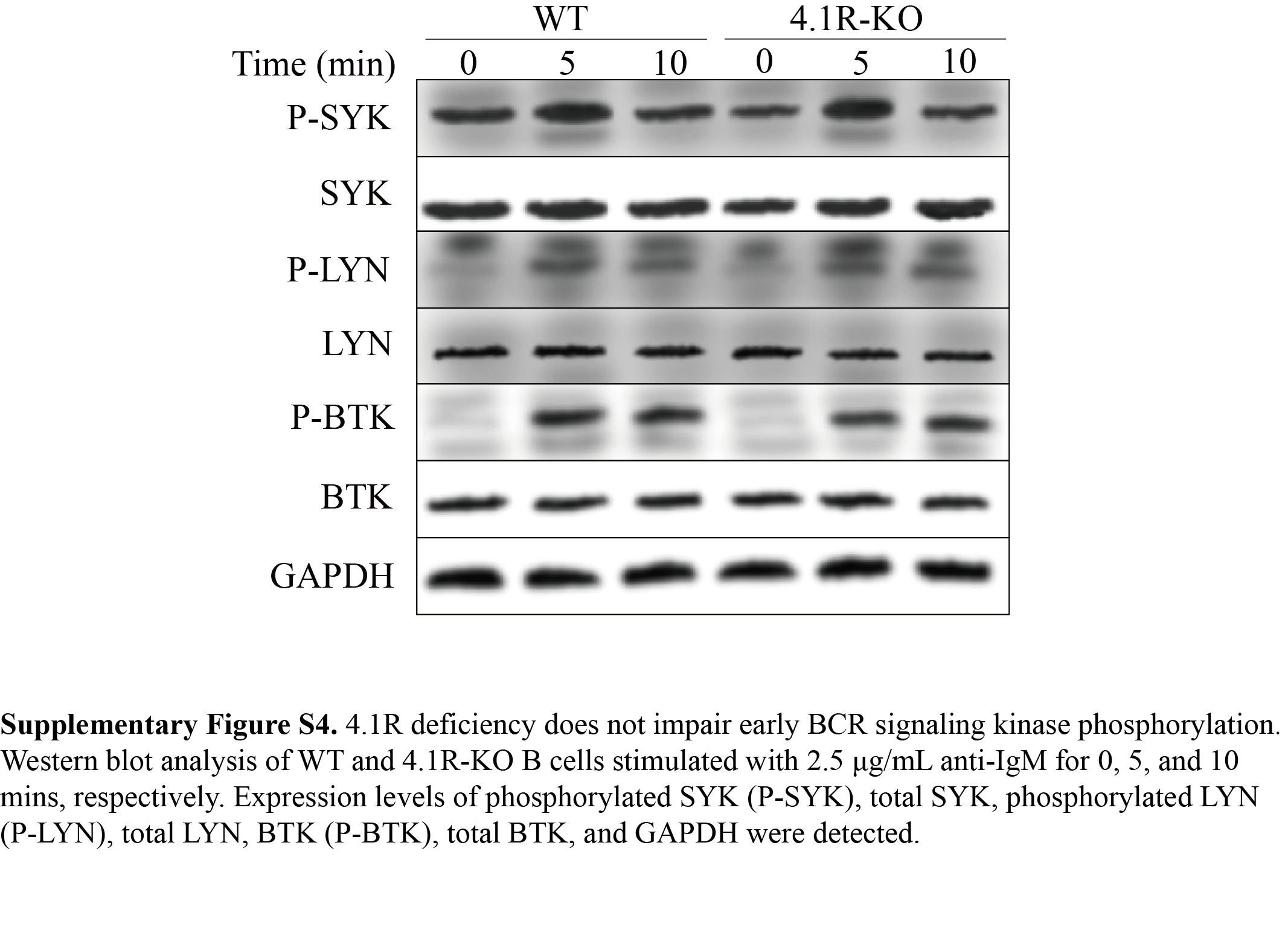

Supplement: Supplementary file 1 [file cells-15-01256-s001.zip › Figure S4.png]
